# Decoupling of Clinical and Laboratory Paradigms: Diagnostic Challenges in Atypical Cytomegalovirus Neurological Immune Reconstitution Inflammatory Syndrome Complicated by Secondary Hemophagocytic Lymphohistiocytosis

**DOI:** 10.7759/cureus.112601

**Published:** 2026-07-13

**Authors:** Jose Hurtado Cordova, Luisa F Vazquez Enriquez, Erik Ovalle, Dahana Rodriguez

**Affiliations:** 1 Internal Medicine, Hospital General de Zona No. 33, Instituto Mexicano del Seguro Social, Monterrey, MEX; 2 Infectious Disease, Hospital General de Zona No. 33, Instituto Mexicano del Seguro Social, Monterrey, MEX

**Keywords:** aki, cmv, corticosteroid therapy, hiv/aids, iris, shlh

## Abstract

The clinical management of patients with advanced human immunodeficiency virus (HIV) infection initiating combination antiretroviral therapy (cART) may be complicated by immune reconstitution inflammatory syndrome (IRIS), a paradoxical inflammatory response capable of unmasking previously subclinical opportunistic infections. Although classical manifestations of IRIS are well recognized, atypical presentations involving the central nervous system (CNS) and associated hyperinflammatory syndromes such as secondary hemophagocytic lymphohistiocytosis (sHLH) remain diagnostically challenging and may contribute to delayed treatment. We report the case of a 36-year-old man with advanced HIV infection (CD4+ T-lymphocyte count of 22.94 cells/µL) who developed persistent fever and neurological symptoms two weeks after initiating cART. Brain magnetic resonance imaging showed no structural abnormalities, and cerebrospinal fluid (CSF) cytochemical analysis was unremarkable. However, a multiplex polymerase chain reaction syndromic panel performed on CSF detected cytomegalovirus (CMV), supporting the diagnosis of unmasking CMV-associated neurological IRIS. During hospitalization, the patient developed a severe systemic inflammatory syndrome characterized by probable secondary hemophagocytic lymphohistiocytosis, stage 3 acute kidney injury, severe cholestatic hepatopathy, and hematologic abnormalities. Targeted antiviral therapy with valganciclovir was initiated promptly, accompanied by a short course of intravenous dexamethasone to control the hyperinflammatory response. The patient experienced progressive clinical improvement with complete recovery of neurological function, normalization of renal function, and marked resolution of hepatic and hematologic abnormalities. This case highlights the diagnostic value of multiplex molecular testing in patients with suspected neuro-IRIS when conventional neuroimaging and CSF studies are non-diagnostic. It also underscores the importance of early recognition and timely combined antiviral and immunomodulatory therapy in severe inflammatory presentations associated with advanced HIV infection.

## Introduction

The initiation of combination antiretroviral therapy (cART) in patients with advanced acquired immunodeficiency syndrome (AIDS), particularly those presenting with CD4+ T-lymphocyte counts below 50 cells/µL, carries a critical risk of developing Immune Reconstitution Inflammatory Syndrome (IRIS) [1]. This immunopathologic phenomenon occurs when the functional restoration of the immune system triggers a dysregulated, hyperinflammatory response against a latent antigen, manifesting as the "unmasking" of a previously subclinical opportunistic infection [2]. Although IRIS is commonly linked to mycobacterial or fungal pathogens, the reactivation of viral agents such as cytomegalovirus (CMV) within the central nervous system (CNS) presents an extraordinarily severe clinical scenario [3].

Central nervous system neuroinfection by CMV as a manifestation of unmasking IRIS represents a profound diagnostic challenge for clinicians, primarily due to the frequent clinicoradiological and cytochemical dissociation exhibited during its initial stages [4]. Classically, CMV encephalitis or ventriculitis presents with cerebrospinal fluid (CSF) pleocytosis and hyperproteinorrachia, accompanied by characteristic periventricular lesions on neuroimaging studies. However, in patients with extreme immunosuppression captured in an early molecular phase, the local inflammatory response may be insufficient to generate macromorphological structural alterations on brain magnetic resonance imaging (MRI) or abnormalities in conventional CSF cytochemical analysis [4,5]. This absence of traditional biomarkers can fatally delay diagnosis, rendering the use of highly sensitive tools-such as multiplex polymerase chain reaction (multiplex PCR) syndromic molecular panels-indispensable for identifying opportunistic viral DNA [5].

The clinical complexity multiplies when the inflammatory storm of IRIS and systemic CMV replication trigger secondary hemophagocytic lymphohistiocytosis (sHLH) [6]. This life-threatening, hyperferritinemic syndrome is driven by severe immune overactivation, specifically the uncontrolled activation of macrophages and cytotoxic T-lymphocytes, leading to massive tissue cytophagia and multiorgan failure [6,7]. While isolated cases of neuro-IRIS are documented in the literature, the simultaneous presentation of unmasking CMV neuro-IRIS complicated by sHLH and multiorgan failure is exceptionally rare, representing a significant gap in current clinical reports. The clinical overlap of severe cytopenias, acute kidney injury (AKI) requiring Stage 3 KDIGO (Kidney Disease: Improving Global Outcomes) classification [8], and a severe cholestatic hepatobiliary pattern can easily mimic bacterial septic shock or drug-induced liver injury (DILI). This introduces a secondary diagnostic conundrum that severely darkens the short-term prognosis unless validated predictive tools, such as the H-Score (a clinical scoring system used to estimate the probability of reactive hemophagocytic syndrome), are promptly implemented [7].

The therapeutic management of this pathogenic triad (neurological IRIS, CMV neuroinfection, and sHLH) constitutes a high-stakes medical dilemma and a complex immunological balancing act [9]. On one hand, definitive treatment for sHLH and IRIS-related inflammation demands the immediate initiation of high-dose systemic corticosteroids (such as dexamethasone) to suppress the lethal cytokine cascade [6,9]. On the other hand, introducing exogenous immunosuppression in a patient with profound AIDS (CD4+ < 25 cells/µL) and an active viral infection in the CNS is a double-edged sword that could theoretically abrogate the host's fragile immune response against CMV, while potentially driving the progression of opportunistic angioproliferative malignancies such as Kaposi Sarcoma [9,10]. The objective of this case report is to describe the clinical success of a dual-targeted therapeutic strategy combining a short course of corticosteroids with targeted valganciclovir therapy dynamically adjusted to the glomerular filtration rate, demonstrating that timely immunomodulation can reverse a catastrophic multiorgan storm.

## Case presentation

A 36-year-old male with no significant medical history presented with a five-month history of a wasting syndrome characterized by chronic cough, involuntary weight loss, night sweats, intermittent fever, and a tension-type headache. Eighteen days prior to hospital admission, an external infectologist confirmed the diagnosis of Human Immunodeficiency Virus (HIV) infection via a positive viral panel, revealing profound immunosuppression with a CD4+ T-lymphocyte count of 22.94 cells/µL (reference range: 500-1,500 cells/µL) and a plasma viral load of 2,510,000 copies/mL (reference range: undetectable). Two weeks prior to admission, cART was initiated with a single-tablet regimen of BIC/FTC/TAF, alongside primary antimicrobial prophylaxis consisting of trimethoprim/sulfametoxazol (80/160 mg/day) and fluconazole (200 mg/day), with no additional screening for opportunistic diseases.

Two weeks after initiating cART, the patient experienced an exacerbation of febrile peaks, chills, and headache, prompting admission to our Internal Medicine service. Upon initial physical examination, the patient was conscious and oriented in all three spheres. A comprehensive neurological examination revealed intact cranial nerves, preserved 5/5 motor strength in all extremities, normal deep tendon reflexes, and no sensory deficits. Furthermore, no signs of meningeal irritation (Kernig or Brudzinski) were detected, and the remainder of the physical examination was otherwise unremarkable. Initial thoracoabdominal computed tomography (CT) demonstrated multiple lymphadenopathies within the para-aortic, interaortocaval, mesenteric root, and ileocolic regions, with no evidence of pulmonary infiltrates or intra-abdominal fluid collections (Figure 1A). An ophthalmologic evaluation ruled out CMV retinitis.

**Figure 1:**
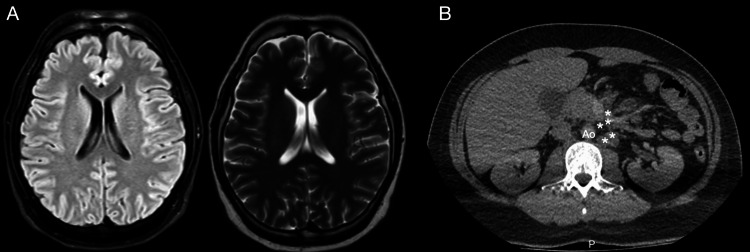
Radiological composite of local and systemic involvement in advanced AIDS. (A) Axial brain MRI utilizing FLAIR (left) and T2-weighted (right) sequences. The brain parenchyma is structurally preserved with an absolute absence of focal lesions, meningeal enhancement, or ventriculitis typical of CMV encephalitis, thereby documenting the clinicoradiological dissociation characteristic of this case during its early molecular phase. (B) Axial abdominopelvic computed tomography (CT). Deep lymph node involvement is evident, characterized by multiple intra-abdominal lymphadenopathies with a marked tendency to coalesce, forming localized conglomerates predominantly within the left para-aortic region. Ao, aorta; *, lymph nodes. AIDS, Acquired Immunodeficiency Syndrome; FLAIR, fluid-attenuated inversion recovery; CMV, cytomegalovirus.

Given the persistence of neurological symptoms following cART initiation in the setting of a high viral antigenic load, an unmasking IRIS was suspected. On hospital day 2, a lumbar puncture was performed; the CSF cytochemical analysis was normal, and a comprehensive microbiological workup successfully ruled out other common opportunistic etiologies. This included a negative cryptococcal antigen test, negative cerebrospinal fluid Venereal Disease Research Laboratory (VDRL) test, negative GeneXpert MTB/RIF (*Mycobacterium tuberculosis*/rifampicin) assay (Cepheid, Sunnyvale, CA, USA), and mycobacterial cultures, as well as negative PCRs for herpes simplex virus 1/2, varicella-zoster virus, and Epstein-Barr virus. This rigorous approach was crucial to narrow the differential diagnosis. However, a multiplex PCR syndromic molecular panel of the CSF returned positive for CMV. This confirmed a diagnosis of CMV CNS neuroinfection in an early or atypical molecular phase, given the absence of classic structural alterations on brain MRI (Figure 1B). Targeted antiviral therapy with valganciclovir was immediately initiated.

During the subsequent clinical course, coinciding with the inflammatory peak of IRIS and CMV viremia, the patient's condition evolved into an acute, reversible multiorgan dysfunction syndrome defined by the concurrent development of sHLH, KDIGO stage 3 AKI, and a severe cholestatic hepatobiliary pattern. The manifestation of sHLH was characterized by persistent fever, clinical hepatomegaly extending 4 cm below the costal margin, hypertriglyceridemia up to 457 mg/dL (reference range: <150 mg/dL), marked hyperferritinemia of 2,596 ng/mL (reference range: 30-400 ng/mL), and severe bicytopenia featuring a hemoglobin nadir of 6.0 g/dL (reference range: 13.5-17.5 g/dL) with a ++ positive direct Coombs test and moderate thrombocytopenia of 39,000/µL (reference range: 150,000-450,000 /µL); this systemic presentation totaled an H-Score of 178 points, conferring a 54-70% diagnostic probability which allowed clinicians to pragmatically omit a bone marrow biopsy for patient safety (Table 1). Concurrently, the renal axis progressed to KDIGO stage 3 AKI, with serum creatinine rising from a baseline of 0.80 mg/dL (reference range: 0.7-1.3 mg/dL) to a zenith of 3.42 mg/dL, urea reaching 165 mg/dL (reference range: 15-40 mg/dL), and a 24-hour urinary protein quantification of 214 mg (reference range: <150 mg/24h), suggesting acute tubular necrosis of prerenal or septic origin.

**Table 1:** H-Score calculation for the non-invasive diagnosis of secondary hemophagocytic lymphohistiocytosis (sHLH). sHLH, secondary hemophagocytic lymphohistiocytosis; AST, aspartate aminotransferase. The H-Score [7] is a validated predictive model used to estimate the probability of reactive hemophagocytic syndrome. In this patient, a cumulative score of 178 points conferred a 54-70% diagnostic probability. This high-probability score pragmatically justified the omission of an invasive bone marrow biopsy in the setting of severe thrombocytopenia (39,000/µL) and elevated hemorrhagic risk, safely facilitating the immediate initiation of life-saving immunomodulatory therapy.

H-Score parameter	Patient's clinical/ laboratory finding	Points assigned
Known underlying immunosuppression	Positive (HIV infection/advanced AIDS)	+ 18
Temperature (°C)	< 38.4 °C at the time of peak multiorgan failure	0
Organomegaly	Clinical hepatomegaly (4 cm below the costal margin)	+ 38
Number of cytopenias	2 lineages (Hemoglobin: 6.0 g/dL; Platelets: 39,000 /µL)	+ 24
Ferritin (ng/mL)	2,596 ng/mL (Criteria: > 2,000)	+ 35
Triglycerides (mg/dL)	457 mg/dL (Criteria: > 135)	+ 44
Fibrinogen (mg/dL)	498 mg/dL (Criteria: ≤ 250)	0
AST (U/L)	177 U/L (Criteria: ≥ 30)	+ 19
Hemophagocytosis on bone marrow	Not performed (Omitted due to high hemorrhagic risk)	0
Total H-Score		178 points
Diagnostic probability		54 - 70%

Finally, severe hepatobiliary involvement was evidenced by a predominantly cholestatic pattern, which correlated chronologically with an abdominal ultrasound (USG) performed on hospital day 4 that demonstrated hepatomegaly with diffusely increased echogenicity and a distended gallbladder containing biliary sludge, notably in the absolute absence of intrahepatic or extrahepatic bile duct dilation. This non-obstructive ultrasonographic presentation, paired with laboratory findings from hospital days 4 and 5 showing a total bilirubin peak of 5.73 mg/dL (reference range: 0.1-1.2 mg/dL), direct bilirubin of 5.35 mg/dL (reference range: 0.0-0.3 mg/dL), alkaline phosphatase of 1,320 U/L (reference range: 44-147 U/L), and GGT of 790 U/L (reference range: 8-61 U/L)(R-factor of 0.2), strongly substantiated an intrahepatic canalicular or infiltrative process, thereby establishing a critical differential diagnosis between HIV-associated cholangiopathy, parenchymal liver infiltration secondary to CMV/sHLH, and DILI.

Due to the pathogenic overlap between neurological IRIS and CMV-associated sHLH, immunomodulatory therapy with intravenous dexamethasone at a dose of 10 mg/m² (16 mg/day) was initiated on hospital day 4 for a short seven-day course. Concurrently, the valganciclovir dose was dynamically adjusted based on the patient's glomerular filtration rate (initially tailored to 450 mg/day, and subsequently optimized to 900 mg every 12 hours as the renal failure resolved). Non-invasive, aggressive fluid management and packed red blood cell transfusions were administered.

The response to the combined antiviral, antiretroviral, and corticosteroid regimen was remarkably favorable. By hospital day 10, the patient was completely afebrile, asymptomatic, and neurologically intact. Biomarkers demonstrated the resolution of the multiorgan failure: serum creatinine normalized to 0.80 mg/dL, total bilirubin decreased to 3.46 mg/dL, and the platelet count rose to 102,000/µL (Figure 2). Over the next few days, the patient continued with excellent oral tolerance and sustained clinical stability; the patient was subsequently discharged to continue outpatient antiviral management and close follow-up by the Infectious Diseases service. During the one-month outpatient follow-up, the patient has achieved robust and continuous clinical resolution across all affected multiorgan axes. He demonstrates excellent adherence to his prescribed therapeutic regimens under the ongoing surveillance of the Infectious Diseases service.

**Figure 2:**
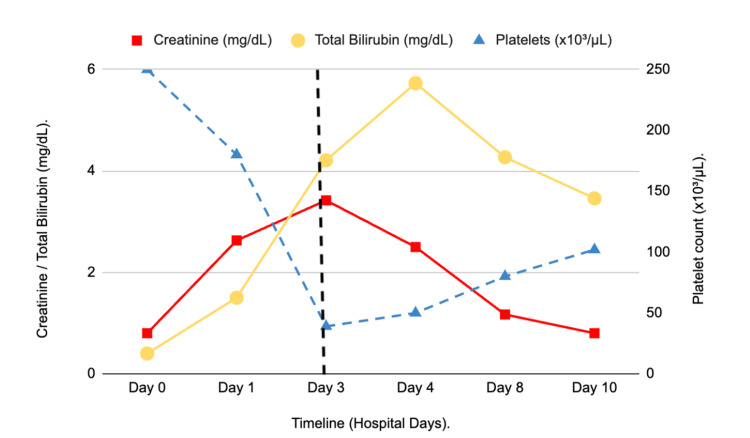
Clinical timeline of multiorgan dysfunction and therapeutic response. This graph provides a consolidated longitudinal summary of the patient's multiorgan evolution over a 10-day hospital course, demonstrating the chronological decoupling of laboratory parameters during the systemic hyperinflammatory peak. It specifically tracks the renal (serum creatinine, red squares), hepatic (total bilirubin, yellow circles), and hematological (platelet count, blue triangles) axes. Notice the synchronous zenith of acute kidney injury (serum creatinine peak: 3.42 mg/dL) and severe cholestatic hepatitis (total bilirubin peak: 5.73 mg/dL) inversely correlating with a profound platelet count nadir (39,000/µL) between hospital days 3 and 4, reflecting the peak of the sHLH storm. The vertical black dotted line indicates the exact initiation of the dual-targeted therapeutic intervention: short-term immunomodulation (intravenous dexamethasone) and dynamically renal-adjusted antiviral therapy (valganciclovir). Following this combined intervention, the chart visually maps the precipitous decline in multi-organ damage biomarkers and the progressive, sustained hematological recovery leading up to hospital discharge on day 10.

## Discussion

The complex clinical scenario presented herein exemplifies a hyperinflammatory tempest arising at the crossroads of advanced HIV infection, opportunistic viral replication, and immune recovery. The initiation of cART in individuals with profound immunosuppression (CD4+ count < 50 cells/µL) is well-documented to precipitate IRIS [1,2]. In this patient, the precipitous collapse of the plasma HIV viral load from 2,510,000 copies/mL to 392 copies/mL within weeks of initiating a single-tablet regimen of BIC/FTC/TAF indicates an exceptionally robust virological response. However, this rapid clearance of viral antigens drove a quantitative and qualitative shift in T-cell dynamics, restoring pathogen-specific immune responses that unmasked a latent, subclinical CMV infection within the CNS [2,3].

From a diagnostic perspective, the presentation of this unmasking neurological CMV-IRIS bypassed classical neurodiagnostic paradigms, exhibiting a profound clinicoradiological and cytochemical dissociation [4]. Standard cytochemical metrics of the CSF-such as glucose, protein, and cell counts-remained paradoxically unremarkable, and brain MRI demonstrated a total absence of macrostructural lesions or periventricular contrast enhancement. Pathophysiologically, in the earliest molecular phases of unmasking neuro-IRIS within an extremely immunocompromised host, the local endothelial recruitment of inflammatory cells may be insufficient to alter conventional CSF parameters or cross the threshold of macromorphological tissue destruction detectable by structural neuroimaging [4,5]. Consequently, relying solely on traditional biomarkers creates a perilous diagnostic latency. This case strongly reinforces the indispensable role of multiplex PCR syndromic panels. Due to its high analytical sensitivity, multiplex PCR successfully amplified CMV DNA within the CSF, allowing clinicians to capture the neuroinfection in an atypical molecular window and initiate targeted antiviral therapy before irreversible parenchymal necrosis ensued [5,11].

The clinical course was further complicated as the localized hyperinflammatory response of IRIS and systemic CMV viremia spiraled into sHLH. In susceptible hosts, active CMV replication serves as a potent driver of pathological macrophage and cytotoxic T-lymphocyte overactivation, triggering an unregulated, self-sustaining systemic cytokine storm [6,9]. The clinical presentation mimicked bacterial septic shock and DILI due to the concurrent development of severe bicytopenia, KDIGO stage 3 AKI, and a severe cholestatic hepatobiliary pattern [7]. Differentiating viral-driven sHLH from profound DILI, HIV-associated cholangiopathy, or overwhelming sepsis is immensely challenging and requires the meticulous exclusion of alternative etiologies. Crucially, abdominal USG demonstrated hepatomegaly and biliary sludge in the absolute absence of intrahepatic or extrahepatic bile duct dilation, confirming that the severe hyperbilirubinemia (total bilirubin peak of 5.73 mg/dL) was driven by an intrahepatic canalicular or infiltrative process secondary to the sHLH systemic storm, rather than a mechanical macrovascular obstructive process. Given the patient's severe bleeding diathesis (platelets at 39,000/µL) and positive direct Coombs test, performing an invasive bone marrow or liver biopsy was clinically contraindicated. This situation highlights the utility of the H-Score as a non-invasive, objective diagnostic tool in the intensive care and internal medicine settings [7]. Generating a score of 178 points provided a 54-70% diagnostic probability of sHLH. Although the absence of definitive histopathological confirmation remains an inherent limitation of this report, relying on such validated predictive metrics and subsequent clinical response successfully bypassed the need for invasive procedures and prevented dangerous diagnostic inertia [6,7].

The management of this overlapping triad (neurological IRIS, active CMV neuroinfection, and sHLH) constituted a high-stakes therapeutic dilemma and a complex immunological balancing act [9]. Suppressing the life-threatening macrophage-mediated multi-organ damage required the immediate initiation of high-dose systemic corticosteroids [6]. However, introducing potent exogenous immunosuppression in a patient with a CD4+ count of 22.94 cells/µL and active viral replication within the CNS represents a dangerous therapeutic paradox [1,9]. The safety and success of this approach depended entirely on simultaneous, robust antiviral coverage. Valganciclovir dosing required aggressive, dynamic titration tailored to the fluctuating glomerular filtration rate during the KDIGO stage 3 AKI phase to prevent subtherapeutic viral under-dosing or ganciclovir-induced myelotoxicity, which would have worsened the pre-existing sHLH cytopenias [12,13]. Furthermore, close clinical vigilance was mandatory due to the risk of triggering opportunistic neoplastic flares, such as a Kaposi Sarcoma flare, induced by corticosteroid-driven upregulation of viral oncogenesis [10]. The favorable outcome of this patient, culminating in complete neurological restoration and normalization of renal and hepatic biomarkers, provides clinical evidence suggesting that short-term immunomodulation may safely mitigate a catastrophic hyperinflammatory storm without compromising viral clearance, provided it is coupled with precise, renal-adjusted targeted antiviral therapy. Nevertheless, as a single case observation, this therapeutic approach cannot be broadly generalized without further cohort validation, underscoring the need for larger studies to solidify management guidelines for this complex triad.

## Conclusions

This case highlights the critical importance of maintaining a high index of suspicion for atypical, multi-organ hyperinflammatory complications when managing advanced AIDS patients undergoing immune restoration. The occurrence of an unmasking neurological CMV-IRIS concurrently driving systemic sHLH underscores that localized viral replication within the CNS can trigger a systemic cytokine storm capable of rapidly progressing to multi-organ failure.

Crucially, this clinical scenario highlights that early molecular identification via syndromic multiplex PCR panels is a highly valuable tool when conventional cytochemical and radiologic paradigms decouple, allowing for targeted therapeutic intervention prior to the onset of irreversible structural damage. Furthermore, our findings support the potential safety and efficacy of an aggressive, dual-targeted management strategy; timely and calculated short-term corticosteroid immunomodulation can successfully suppress a life-threatening cytokine storm without compromising viral clearance, provided it is supported by robust, dynamically titrated, renal-adjusted antiviral coverage.
